# Supplementary material for: Structural and molecular rationale for the diversification of resistance mediated by the Antibiotic_NAT family
Source: Commun Biol. 2022 Mar 25;5:263. doi: 10.1038/s42003-022-03219-w (PMC8956665; doi:10.1038/s42003-022-03219-w)
Supplement: Supplementary file 2 — Supplementary Information [file 42003_2022_3219_MOESM2_ESM.pdf]

<u>**Supplementary Information**</u>

Structural and molecular rationale for the diversification of resistance mediated by the Antibiotic_NAT family

Peter J Stogios[1,#], Emily Bordeleau[2,#], Zhiyu Xu[1], Tatiana Skarina[1], Elena Evdokimova[1], Sommer Chou[2], Luke Diorio-Toth[3], Alaric W. D'Souza[3], Sanket Patel[3], Gautam Dantas[3,4,5,6], Gerard D. Wright[2] and Alexei Savchenko[1,7,8,*]

[1]Department of Chemical Engineering and Applied Chemistry, University of Toronto, Toronto, Canada

[2]David Braley Centre for Antibiotics Discovery M.G. DeGroote Institute for Infectious Disease Research, Department of Biochemistry and Biomedical Sciences, McMaster University, Hamilton, ON, Canada

[3]The Edison Family Center for Genome Sciences & Systems Biology, Washington University School of Medicine, St. Louis, MO, USA, 63108

[4]Department of Pathology and Immunology, Washington University School of Medicine, St. Louis, MO, USA, 63108

[5]Department of Biomedical Engineering, Washington University in St. Louis, St. Louis, MO, USA, 63130

[6]Department of Molecular Microbiology, Washington University School of Medicine, St. Louis, MO, USA, 63108

[7]Department of Microbiology, Immunology and Infectious Diseases, University of Calgary, Calgary, Canada, T2N 4N1.

[8]Center for Structural Genomics of Infectious Diseases (CSGID), University of Calgary, Calgary, Canada, T2N 4N1.

[*]to whom correspondence should be addressed: Alexei Savchenko, Department of Immunology and Infectious Diseases, Health Research Innovation Centre, 3330 Hospital Drive NW, University of Calgary, Calgary, AB, T2N 4N1, Canada. Tel: +1-403-210-7980; Email: alexei.savchenko@ucalgary.ca

[#]These authors contributed equally

Supplementary Figure 1 – Sequence alignment of the Antibiotic_NAT family.

Sequences are grouped and shaded according to phylogenetic reconstruction in Figure 1. Includes meta-AAC's, AAC(3) enzymes from clinical isolates, and sequences identified through BLAST searches of NCBI. Darker color or shading of amino acids indicates higher conservation. Major and minor subdomains are indicated with solid and dashed black lines, respectively, above the sequence alignment.

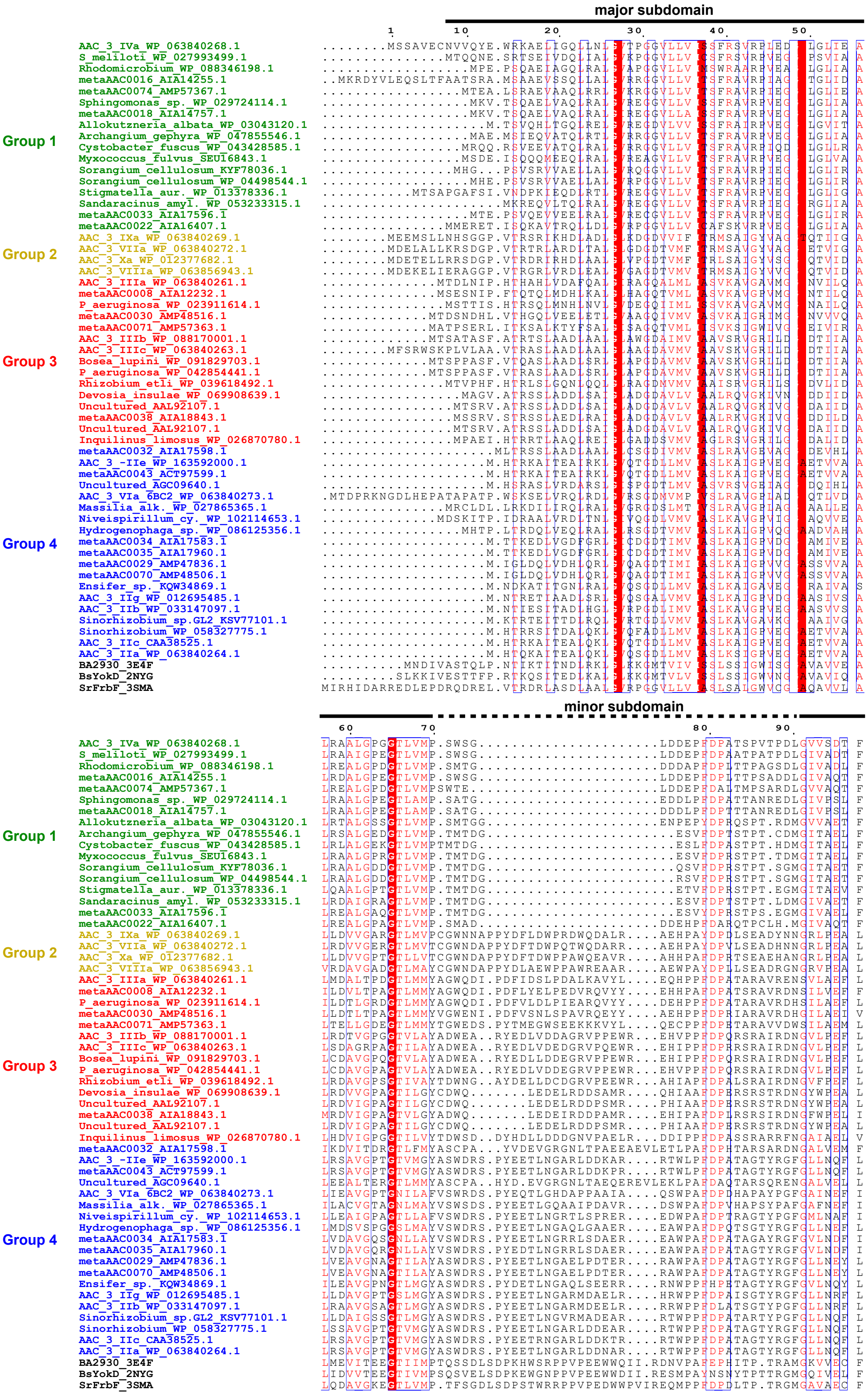

major subdomain
minor subdomain
Group 1
Group 2
Group 3
Group 4
AAC_3_IVa_WP_063840268.1
S_meliloti_WP_027993499.1
Rhodomicrobium_WP_088346198.1
metaAAC0016_AIA14255.1
metaAAC0074_AMP57367.1
Sphingomonas_sp._WP_029724114.1
metaAAC0018_AIA14757.1
Allokutzneria_albata_WP_03043120.1
Archangium_gephyra_WP_047855546.1
Cystobacter_fuscus_WP_043428585.1
Myxococcus_fulvus_SEU16843.1
Sorangium_cellulosum_KYF78036.1
Sorangium_cellulosum_WP_04498544.1
Stigmatella_aur._WP_013378336.1
Sandaracinus_amyl._WP_053233315.1
metaAAC0033_AIA17596.1
metaAAC0022_AIA16407.1
AAC_3_IXa_WP_063840269.1
AAC_3_VIIa_WP_063840272.1
AAC_3_Xa_WP_012377682.1
AAC_3_VIIIa_WP_063856943.1
AAC_3_IIIa_WP_063840261.1
metaAAC0008_AIA12232.1
P_aeruginosa_WP_023911614.1
metaAAC0030_AMP48516.1
metaAAC0071_AMP57363.1
AAC_3_IIIb_WP_088170001.1
AAC_3_IIIc_WP_063840263.1
Bosea_lupini_WP_091829703.1
P_aeruginosa_WP_042854441.1
Rhizobium_etli_WP_039618492.1
Devosia_insulae_WP_069908639.1
Uncultured_AAL92107.1
metaAAC0038_AIA18843.1
Uncultured_AAL92107.1
Inquilinus_limosus_WP_026870780.1
metaAAC0032_AIA17598.1
AAC_3_-IIe_WP_163592000.1
metaAAC0043_ACT97599.1
Uncultured_AGC09640.1
AAC_3_VIa_6BC2_WP_063840273.1
Massilia_alk._WP_027865365.1
Niveispirillum_cy._WP_102114653.1
Hydrogenophaga_sp._WP_086125356.1
metaAAC0034_AIA17583.1
metaAAC0035_AIA17960.1
metaAAC0029_AMP47836.1
metaAAC0070_AMP48506.1
Ensifer_sp._KQW34869.1
AAC_3_IIg_WP_012695485.1
AAC_3_IIb_WP_033147097.1
Sinorhizobium_sp.GL2_KSV77101.1
Sinorhizobium_WP_058327775.1
AAC_3_IIc_CAA38525.1
AAC_3_IIa_WP_063840264.1
BA2930_3E4F
BsYokD_2NYG
SrFrbF_3SMA

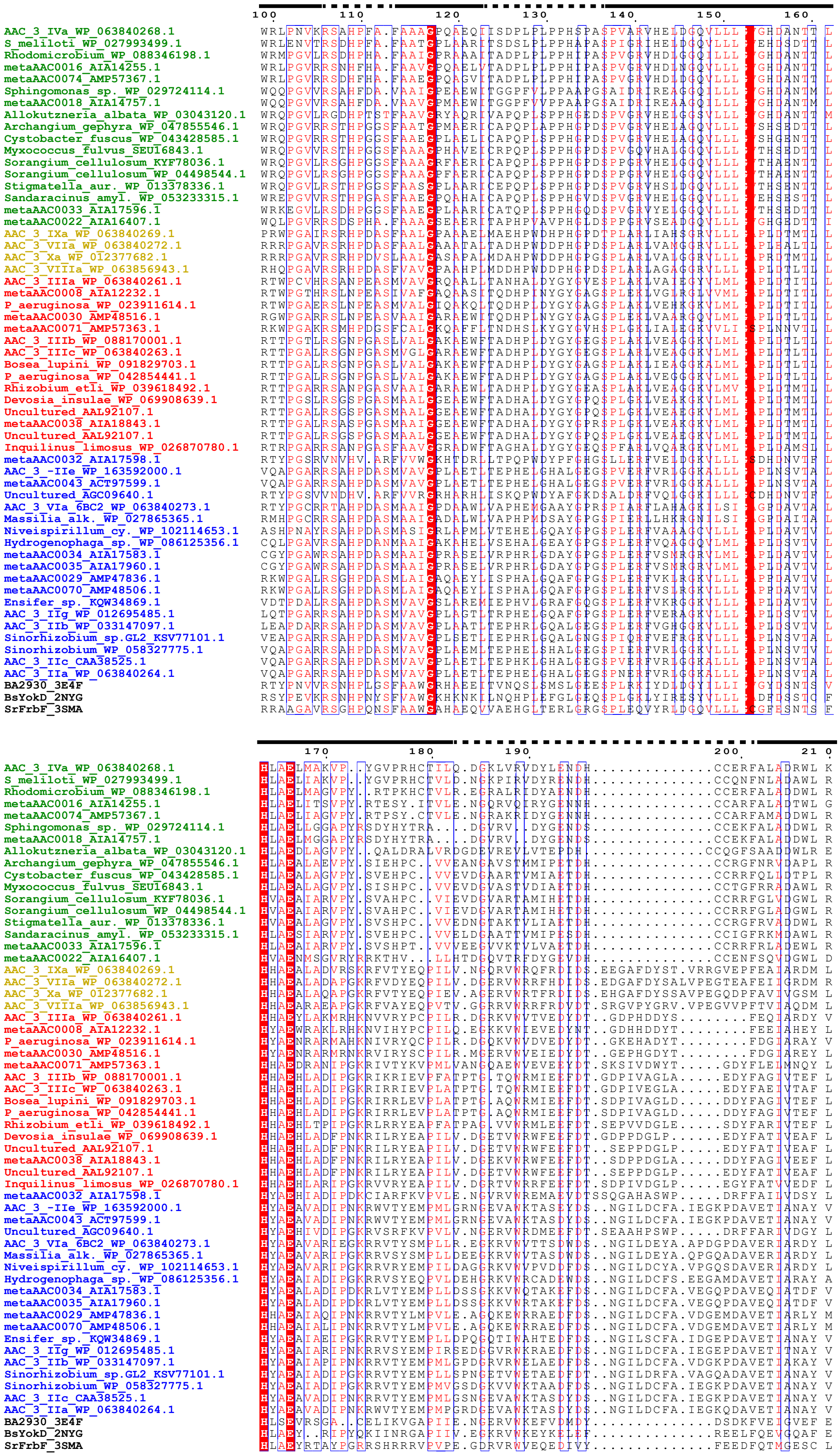
100 110 120 130 140 150 160
AAC_3_IVa_WP_063840268.1
S_meliloti_WP_027993499.1
Rhodomicrobium_WP_088346198.1
metaAAC0016_AIA14255.1
metaAAC0074_AMP57367.1
Sphingomonas_sp._WP_029724114.1
metaAAC0018_AIA14757.1
Allokutzneria_albata_WP_03043120.1
Archangium_gephyra_WP_047855546.1
Cystobacter_fuscus_WP_043428585.1
Myxococcus_fulvus_SEU16843.1
Sorangium_cellulosum_KYF78036.1
Sorangium_cellulosum_WP_04498544.1
Stigmatella_aur._WP_013378336.1
Sandaracinus_amyl._WP_053233315.1
metaAAC0033_AIA17596.1
metaAAC0022_AIA16407.1
AAC_3_IXa_WP_063840269.1
AAC_3_VIIa_WP_063840272.1
AAC_3_Xa_WP_012377682.1
AAC_3_VIIIa_WP_063856943.1
AAC_3_IIIa_WP_063840261.1
metaAAC0008_AIA12232.1
P_aeruginosa_WP_023911614.1
metaAAC0030_AMP48516.1
metaAAC0071_AMP57363.1
AAC_3_IIIb_WP_088170001.1
AAC_3_IIIc_WP_063840263.1
Bosea_lupini_WP_091829703.1
P_aeruginosa_WP_042854441.1
Rhizobium_etli_WP_039618492.1
Devosia_insulae_WP_069908639.1
Uncultured_AAL92107.1
metaAAC0038_AIA18843.1
Uncultured_AAL92107.1
Inquilinus_limosus_WP_026870780.1
metaAAC0032_AIA17598.1
AAC_3_-IIe_WP_163592000.1
metaAAC0043_ACT97599.1
Uncultured_AGC09640.1
AAC_3_VIa_6BC2_WP_063840273.1
Massilia_alk._WP_027865365.1
Niveispirillum_cy._WP_102114653.1
Hydrogenophaga_sp._WP_086125356.1
metaAAC0034_AIA17583.1
metaAAC0035_AIA17960.1
metaAAC0029_AMP47836.1
metaAAC0070_AMP48506.1
Ensifer_sp._KQW34869.1
AAC_3_IIg_WP_012695485.1
AAC_3_IIb_WP_033147097.1
Sinorhizobium_sp.GL2_KSV77101.1
Sinorhizobium_WP_058327775.1
AAC_3_IIc_CAA38525.1
AAC_3_IIa_WP_063840264.1
BA2930_3E4F
BsYokD_2NYG
SrFrbF_3SMA
170 180 190 200 210

```
                                                    220        230        240        250        260
AAC_3_IVa_WP_063840268.1             EKSLQK.EGPVGHAFARLIRSRDIVATALGQLGRDPLIFLHPPEAGCEECDAARQSIG...... .
S_meliloti_WP_027993499.1            REGLQR.EGPVGHAHARLIRSRDIVRIASDRLARDPLVFLHPIEAACEECDAARQSAGCAPRST S
Rhodomicrobium_WP_088346198.1        ARGLQS.EGRVGRAHARLFRARHVTEAALGHLAAEPLLFLHRAGAHCPACDAARQSTSG..... .
metaAAC0016_AIA14255.1               DAGRQA.EGQVGHAHARLADARDIVALAVERLVADPLLFLHAPEAGCPECVEARQSVSVGEPIR N
metaAAC0074_AMP57367.1               ARGLQT.EARVGQADARLSRSCDIVSVALEYLSMNPLLFLHPVGAGCDECDEARQTAGAAPFIK N
Sphingomonas_sp._WP_029724114.1      AEGLQC.EGRIGNADSRLFDVEDLIRVAVPRVQADPLVFLHGSE.GCEECDEARESLSR..... .
metaAAC0018_AIA14757.1               AEGLQR.EGRIGNAHARLIEAEDLIRVAVPRLRADPLVFLHGADQGCEECGEARESLSR..... .
Allokutzneria_albata_WP_03043120.1   DGAQR.EGRVGNAEARLFAARDLVRTVVPRLTADPLRFLCAEGSGCEDCDDARRSARHP..... .
Archangium_gephyra_WP_047855546.1    ARGLQH.EGLVGHAQARLCDSRDIVSVALGLLAPDPLVFLCPPEEHCEECDQARASVKTRGGVR N
Cystobacter_fuscus_WP_043428585.1    ARALQH.EGLVGHALARLCDSRDVVAVALELLASEPLLFLCPPEEHCEECDVARASLRAT.... .
Myxococcus_fulvus_SEU16843.1         ARGLQR.EGKVGHADARLADARDIVAVAMEQLATDPLVFLCGPDAGCEECTLARDSVSR..... .
Sorangium_cellulosum_KYF78036.1      GRGLQQ.EGRVGSAHARLCASRDLVAVAVEHLAKSPLVFLCPPDEGCDECDAARASVPADAARG E
Sorangium_cellulosum_WP_04498544.1   ERGLQQ.EGRVGRAHARVCASRDLVAVAVEHLAKSPLVFLCPPDEGCDECDAARASVPAGGARR E
Stigmatella_aur._WP_013378336.1      PRGLQR.EGQVGNAHARLCNARDLVRLAVEHLAADPLTFLCAPETGCEECDAAHASIPAGVRR. .
Sandaracinus_amyl._WP_053233315.1    ARDLQR.EGPVGSAHARLCDARDVVRIALERLASDPLTFLCGAGARCEECDRARASIAAE.... .
metaAAC0033_AIA17596.1               AKGLQR.EGKVGSATARLADAKHIVAVAVERLAADPIVFLCPPSEACEECDLARASIK...... .
metaAAC0022_AIA16407.1               AQSMQR.RGKVGHADARLARSRDIIAVASAQLRENETVFLHPPGIDA.ECDEARASIPQES... .
AAC_3_IXa_WP_063840269.1             SAGIGR.QGRVGAADSYLFDAGPVFNFAINWI...EAKLKR....................... .
AAC_3_VIIa_WP_063840272.1            AAGIGR.RGTVGAADSHLFEARDVVDFGVAWM...EEKLGRERGPGG................. .
AAC_3_Xa_WP_012377682.1              AAGIGR.EGFVGAARSRLFDAAPAVEFGVRWI...EEHLNRDR..................... .
AAC_3_VIIIa_WP_063856943.1           AAGIGR.TGRVAAAPVHLFEAADVVRFGVEWI...ESRMGGAAGGA.................. .
AAC_3_IIIa_WP_063840261.1            AQGGGT.RGKVGDADAYLFAAQDLTRFAVQWL...ESRFGDSASYG.................. .
metaAAC0008_AIA12232.1               AQGKTS.HGTVGDADTYVFDAADLSEFAVQWL...EARFGA....................... .
P_aeruginosa_WP_023911614.1          AEGRGT.QGSVGNAESYLFDAADLAAFAITWL...EVRFG........................ .
metaAAC0030_AMP48516.1               AQGKGR.QGTIGHAPSYLFDAADLSTFAIAWL...EERFGETT..................... .
metaAAC0071_AMP57363.1               ADFKIV.SRKVGNARSYLLDAAHLNSYARAWM...EKHLSRRPD.................... .
AAC_3_IIIb_WP_088170001.1            ASGQGR.QGLIGAAPSVLVDAAAITAFGVTWL...EKRFGTPSP.................... .
AAC_3_IIIc_WP_063840263.1            AGGRGR.QGLIGTAPSVLVDAAAITAFGVAWL...ESRFGSPSS.................... .
Bosea_lupini_WP_091829703.1          AAGEGR.QGLIGAAHSVLVEAAAITAFGVDWL...ESRFGSS...................... .
P_aeruginosa_WP_042854441.1          AAGEGR.QGLIGAAHSVLVEAAAITAFGVDWL...ESRFGSSSLRL.................. .
Rhizobium_etli_WP_039618492.1        AGGQGA.QGPIGEAPSVLVDAASICSFAVAWL...EQHVQPEN..................... .
Devosia_insulae_WP_069908639.1       ATGRGK.RGKVGEANSVLVPAPEVVAFGVDWL...E.RWGKGL..................... .
Uncultured_AAL92107.1                ATGRGK.RGEVGEASSVLVPAAAMVAFGVDWL...E.RWGKTL..................... .
metaAAC0038_AIA18843.1               ATGRGK.RGKIGEASSVLVPADEIVAFAVDWL...E.RWGRTARR................... .
Uncultured_AAL92107.1                ATGRGK.RGEVGEASSVLVPAAAMVAFGVDWL...E.RWGKTL..................... .
Inquilinus_limosus_WP_026870780.1    AAGGGA.RGTIGAAPSVLVPAAEMADFAVRWL...ESRFGDPQRSGAASV.............. .
metaAAC0032_AIA17598.1               AATNNR.GGRVGRAESYLFSARGLLEHALPKM...EAIARPRR..................... .
AAC_3_-IIe_WP_163592000.1            KLGRHR.EGVVGFAQCYLFDAQDIVTFGVTYL...EKHFGATPIVPAHEAAQRSCEPSG..... .
metaAAC0043_ACT97599.1               KLGRHR.EGVVGFAQCYLFDAQDIVTFGVTYL...EKHFGATPIVPAHEAAQRSCEPSG..... .
Uncultured_AGC09640.1                EKTNNR.GGVLGDSQSYVIDARGLLEHALQEM...KRQAT........................ .
AAC_3_VIa_6BC2_WP_063840273.1        ARTRVA.QGPVGGAQSRLIDAADIVSFGIEWL...EARHAAPAAAALKPKQRRD.......... .
Massilia_alk._WP_027865365.1         ALGRHR.EGQVGRAVARLIDANDIVRFGQAWL...ERRHVNRK..................... .
Niveispirillum_cy._WP_102114653.1    GEKRHR.TGRIGNADSYLLSAPDLVRYGIDWL...ERRHPSV...................... .
Hydrogenophaga_sp._WP_086125356.1    SEGRHR.TGRVGRADCHLFDAQDIVAFGKGWL...EARFR........................ .
metaAAC0034_AIA17583.1               RLGRCR.QGRVANADCFLFDAVQLVDFGVRWL...ERRFGATRQGSRAG............... .
metaAAC0035_AIA17960.1               RLGRYR.QGRVANADCFLFDAVQLVDFGVRWL...ERRFGATRQGSRAG............... .
metaAAC0029_AMP47836.1               SEGHAR.SGRIGAANSYLIEAERIVQYGVRWL...ERLFGRNGSLLPEK............... .
metaAAC0070_AMP48506.1               SEGHAR.SGRIGAANSYLIEAERIVQYGVRWL...ERLFGRNGSLLPEK............... .
Ensifer_sp._KQW34869.1               LLGRHR.EGPVARAHSYLFDAKDIVEFGVEYL...ERNFGAAGLT................... .
AAC_3_IIg_WP_012695485.1             ELRRHR.EGLVGQAHCYLFEARDIVSFGVDYL...QRHFGSP...................... .
AAC_3_IIb_WP_033147097.1             ELGRHR.EGIVGRAPSYLFEAQDIVSFGVTYL...EQHFGAP...................... .
Sinorhizobium_sp.GL2_KSV77101.1      ATSRHR.SGTVGDAFCHLFDARDIVSFGVTYL...ESRFGKGA..................... .
Sinorhizobium_WP_058327775.1         KLGRHR.GDIVGCAQCHLFDAQDIVSFGVTYL...EQHFGTAPLMSAYEPAERSCEPSG..... .
AAC_3_IIc_CAA38525.1                 KLGRHR.EGVVGFAQCYLFDAQDIVTFGVTYL...EKHFGTTPIVPAHEVAECSCEPSG..... .
AAC_3_IIa_WP_063840264.1             KLGRHR.EGVVGFAQCYLFDAQDIVTFGVTYL...EKHFGTTPIVPAHEAIERSCEPSG..... .
BA2930_3E4F                          QKGTVT.MGKIGNAKCRLMKQRDIVDFGTEWF...RKKN......................... .
BsYokD_2NYG                          AEHNMK.VGKVGSANCRLFSLTEAVDFAEKWFINNDSKNIKK...................... .
SrFrbF_3SMA                          TRTPGHSRGTVGEAAAVLYGQRAFVDLACEWM...TAHRDLARAVGA................. .

AAC_3_IVa_WP_063840268.1             .....
S_meliloti_WP_027993499.1            GGGQF
Rhodomicrobium_WP_088346198.1        .....
metaAAC0016_AIA14255.1               MG...
metaAAC0074_AMP57367.1               TG...
Sphingomonas_sp._WP_029724114.1      .....
metaAAC0018_AIA14757.1               .....
Allokutzneria_albata_WP_03043120.1   .....
Archangium_gephyra_WP_047855546.1    TGPA.
Cystobacter_fuscus_WP_043428585.1    .....
Myxococcus_fulvus_SEU16843.1         .....
Sorangium_cellulosum_KYF78036.1      HA...
Sorangium_cellulosum_WP_04498544.1   HA...
Stigmatella_aur._WP_013378336.1      .....
Sandaracinus_amyl._WP_053233315.1    .....
metaAAC0033_AIA17596.1               .....
metaAAC0022_AIA16407.1               .....
AAC_3_IXa_WP_063840269.1             .....
AAC_3_VIIa_WP_063840272.1            .....
AAC_3_Xa_WP_012377682.1              .....
AAC_3_VIIIa_WP_063856943.1           .....
AAC_3_IIIa_WP_063840261.1            .....
metaAAC0008_AIA12232.1               .....
P_aeruginosa_WP_023911614.1          .....
metaAAC0030_AMP48516.1               .....
metaAAC0071_AMP57363.1               .....
AAC_3_IIIb_WP_088170001.1            .....
AAC_3_IIIc_WP_063840263.1            .....
Bosea_lupini_WP_091829703.1          .....
P_aeruginosa_WP_042854441.1          .....
Rhizobium_etli_WP_039618492.1        .....
Devosia_insulae_WP_069908639.1       .....
Uncultured_AAL92107.1                .....
metaAAC0038_AIA18843.1               .....
Uncultured_AAL92107.1                .....
Inquilinus_limosus_WP_026870780.1    .....
metaAAC0032_AIA17598.1               .....
AAC_3_-IIe_WP_163592000.1            .....
metaAAC0043_ACT97599.1               .....
Uncultured_AGC09640.1                .....
AAC_3_VIa_6BC2_WP_063840273.1        .....
Massilia_alk._WP_027865365.1         .....
Niveispirillum_cy._WP_102114653.1    .....
Hydrogenophaga_sp._WP_086125356.1    .....
metaAAC0034_AIA17583.1               .....
metaAAC0035_AIA17960.1               .....
metaAAC0029_AMP47836.1               .....
metaAAC0070_AMP48506.1               .....
Ensifer_sp._KQW34869.1               .....
AAC_3_IIg_WP_012695485.1             .....
AAC_3_IIb_WP_033147097.1             .....
Sinorhizobium_sp.GL2_KSV77101.1      .....
Sinorhizobium_WP_058327775.1         .....
AAC_3_IIc_CAA38525.1                 .....
AAC_3_IIa_WP_063840264.1             .....
BA2930_3E4F                          .....
BsYokD_2NYG                          .....
SrFrbF_3SMA                          .....
```

Supplementary Figure 2 – Structural analysis of Group 4 AAC(3)-IIb vs. AAC(3)-VIa.

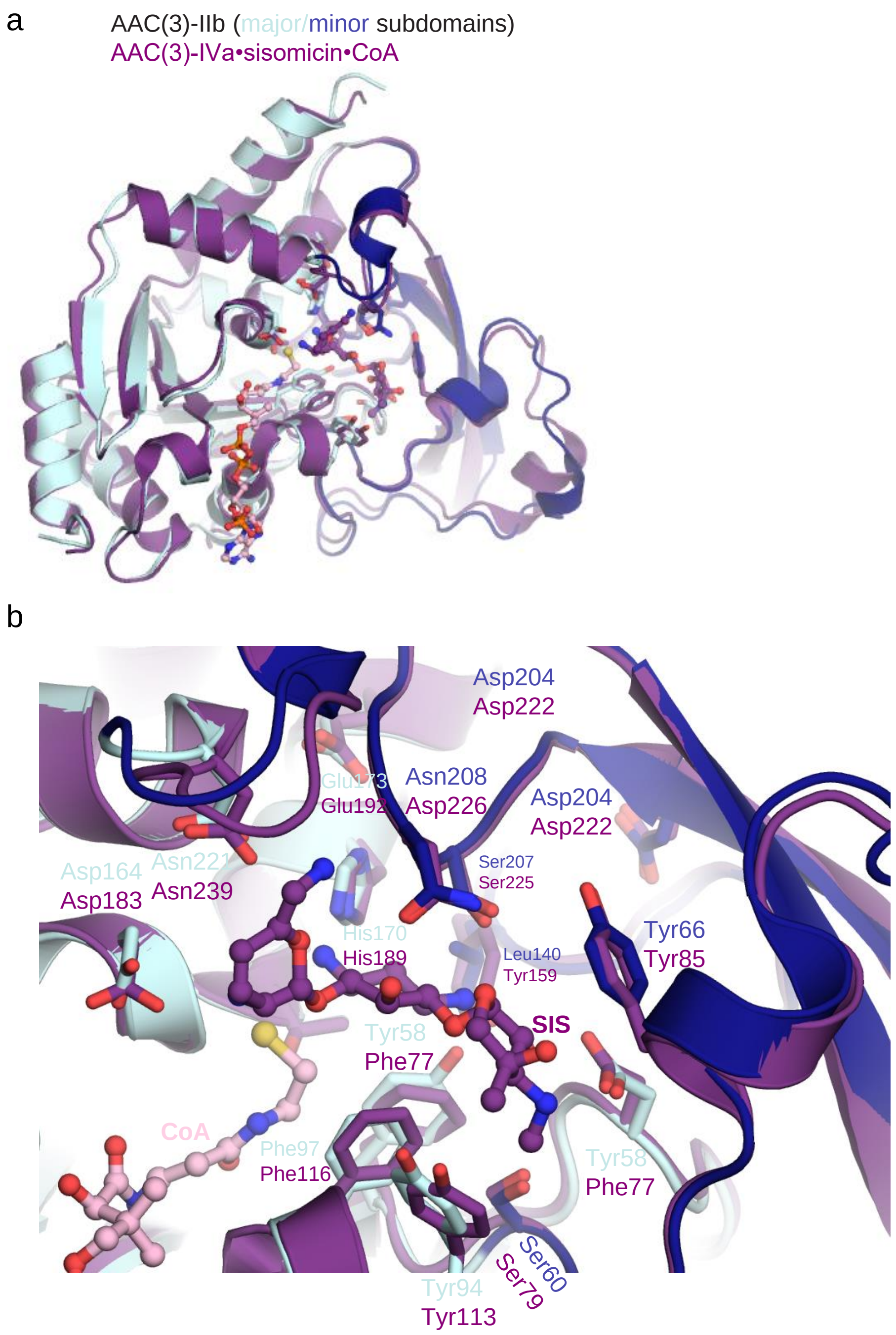

a) Superposition of overall structures. b) Zoom of active sites, sisomicin and CoA are shown in ball-and-stick representation.

Supplementary Table 1. Aminoglycoside susceptibility of *E. coli* harboring Antibiotic_NAT genes

E. coli BW25113 Δ*tolC*Δ*bamB* expressing individual Antibiotic_NAT genes under the control of the P_{bla} promoter in vector pGDP3. Shown are MIC values in µg/mL grouped and coloured as in Figure 2. Raw data used to derive this table is shown in **Supplementary Data 1.**

Group 1

	Control	Meta-AAC0016	Meta-AAC0018	HMB0022	HMB00033	AAC(3)-IVa
**APR**	4	> 256	> 256	> 256	> 256	> 512
**TOB**	0.5	> 256	> 256	> 256	> 256	≥ 64
**GEN**	0.25-0.5	≥ 256	128-256	64	≥ 256	> 512
**KAN**	2	128-256	64-128	8	32	64
**AMI**	1-2	1-2	1	1-2	1	1
**NEO**	1	64	128	8-16	64-128	64-128
**PAR**	2	> 256	128	16	64-128	128-256

Group 2

	AAC(3)-VIIa	AAC(3)-VIIIa	AAC(3)-IXa	AAC(3)-Xa
**APR**	ND	ND	4	2-4
**TOB**	ND	ND	≤ 0.5	16-32
**GEN**	ND	ND	≤ 0.5	8
**KAN**	ND	ND	2	64-128
**AMI**	ND	ND	1	1
**NEO**	ND	ND	1	1
**PAR**	ND	ND	2	4

Group 3

	Meta-AAC0008	Meta-AAC0030	Meta-AAC0038	Meta-AAC0071	AAC(3)-IIIa	AAC(3)-IIIc	AAC(3)-IIIb
**APR**	16-32	16	32-64	4-8	8-16	ND	8
**TOB**	> 256	> 256	> 256	> 256	> 256	ND	> 256
**GEN**	> 256	> 256	> 256	> 256	> 256	ND	> 256
**KAN**	> 256	> 256	> 256	> 256	> 256	ND	> 256
**AMI**	1	1-2	1-2	1-2	1	ND	0.5 – 1
**NEO**	> 256	64-128	256	1	128-256	ND	256
**PAR**	> 256	> 256	> 256	4	> 256	ND	> 256

Group 4

	Meta-AAC0029	Meta-AAC0032	Meta-AAC0035	Meta-AAC0043	Meta-AAC0070	AAC(3)-IIa	AAC(3)-IIb	AAC(3)-IIc	AAC(3)-VIa	Meta-AAC0034
**APR**	4	2-4	8	4	ND	8	16-32	2	4	8
**TOB**	64	4-8	> 256	32-64	ND	> 64	> 256	64 - 128	8	> 256
**GEN**	> 64	64	> 256	≥ 256	ND	> 64	> 256	> 256	≥ 256	> 64
**KAN**	16	16-32	≥ 256	16	ND	>64	> 256	32	16	> 256
**AMI**	0.5-1	1-2	1	1-2	ND	2	1	0.5-1	1-2	1-2
**NEO**	≤ 1	1	1	1	ND	1	0.5-1	0.5-1	≤ 1	≤ 1
**PAR**	1	2	2	2-4	ND	2-4	1-2	0.5-1	2	1

ND = No data.
