# Supplementary material for: Structural and molecular rationale for the diversification of resistance mediated by the Antibiotic_NAT family
Source: Commun Biol. 2022 Mar 25;5:263. doi: 10.1038/s42003-022-03219-w (PMC8956665; doi:10.1038/s42003-022-03219-w)
Supplement: Supplementary file 3 — Description of Additional Supplementary Files [file 42003_2022_3219_MOESM3_ESM.pdf]

Description of Additional Supplementary Files

File name: Supplementary Data 1
Description: Antimicrobial susceptibility testing data for *E. coli* BW25113 ΔtolCΔbamB expressing individual Antibiotic_NAT genes under the control of the Pbla promoter in vector pGDP3.
